# Current clinical practice in adapted automated peritoneal dialysis (aAPD)—A prospective, non-interventional study

**DOI:** 10.1371/journal.pone.0258440

**Published:** 2021-12-09

**Authors:** Manel Vera, Bee Boon Cheak, Hana Chmelíčková, Sunita Bavanandan, Bak Leong Goh, Abdul Gafor Abdul Halim, Isabel Garcia, Martin Gajdoš, Rafael Alonso Valente, Tatiana De los Ríos, Saynab Atiye, Manuela Stauss-Grabo, Emilio Galli

**Affiliations:** 1 Nephrology, Hospital Clinic de Barcelona, Barcelona, Spain; 2 Department of Nephrology, Hospital Selayang, Selangor, Malaysia; 3 Nephrology, Hospital Trebic, Trebic, Czech Republic; 4 Nephrology, Hospital Kuala Lumpur, Kuala Lumpur, Malaysia; 5 Department of Nephrology & Clinical Research Centre, Hospital Serdang, Selangor, Malaysia; 6 Universiti Kebangsaan Malaysia, Kuala Lumpur, Malaysia; 7 Nephrology, Hospital Universitario di Girona Josep Trueta, Girona, Spain; 8 Nephrology, NC Centre Sokolov, Sokolov, Czech Republic; 9 Nephrology, Complejo Hospital Universitario de Santiago, Santiago de Compostela, Spain; 10 Fresenius Medical Care, Global Medical Office, Bad Homburg, Germany; 11 Nefrologia e dialisi, ASST Bergamo Ovest, Treviglio, Italy; Istituto Di Ricerche Farmacologiche Mario Negri, ITALY

## Abstract

Adapted automated peritoneal dialysis (aAPD), comprising a sequence of dwells with different durations and fill volumes, has been shown to enhance both ultrafiltration and solute clearance compared to standard peritoneal dialysis with constant time and volume dwells. The aim of this non-interventional study was to describe the different prescription patterns used in aAPD in clinical practice and to observe outcomes characterizing volume status, dialysis efficiency, and residual renal function over 1 year. Prevalent and incident, adult aAPD patients were recruited during routine clinic visits, and aAPD prescription, volume status, residual renal function and laboratory data were documented at baseline and every quarter thereafter for 1 year. Treatments were prescribed according to the nephrologist’s medical judgement in accordance with each center’s clinical routine. Of 180 recruited patients, 160 were analyzed. 27 different aAPD prescription patterns were identified. 79 patients (49.4%) received 2 small, short dwells followed by 3 long, large dwells. During follow-up, volume status changed only marginally, with visit mean values ranging between 1.59 (95% confidence interval: 1.19; 1.99) and 1.97 (1.33; 2.61) L. Urine output and creatinine clearance decreased significantly, accompanied by reductions in ultrafiltration and Kt/V. 25 patients (15.6%) received a renal transplant and 15 (9.4%) were changed to hemodialysis. Options for individualization offered by aAPD are actually used in practice for optimized treatment. Changes observed in renal function and dialysis efficiency measures reflect the natural course of chronic kidney disease. No safety events were observed during the study period.

## Introduction

Volume overload is a common problem in patients undergoing hemodialysis (HD) or peritoneal dialysis (PD) [1]. It is encountered on both incident and prevalent patients, indicating that patients with an indication for renal replacement therapy tend to be already overloaded when they start dialysis [2]. In PD, volume overload has been associated with poorer outcome, technique failure, and ultimately with increased mortality [3, 4]. Volume control is therefore both an important therapeutic target in PD and a clinically relevant outcome when investigating the effectiveness of dialysis treatment.

In the last decades, several attempts towards an optimization of automated peritoneal dialysis (APD) have been made in order to provide better fluid and sodium removal. Major progress in this regard has been suggested by a concept called adapted automated peritoneal dialysis (aAPD), which was investigated for the first time by Fischbach et al. in a pilot trial in children published in 1994 [5]. Their approach is based on the observation that fill volume and dwell time impact the ultrafiltration and clearance achieved by PD treatment significantly. When combining 2 short dwells with low volume, enhancing ultrafiltration, with 3 subsequent long dwells with high volume, enhancing solute clearance, they observed both more efficient ultrafiltration with lower glucose absorption and improved sodium and uremic toxin removal compared to conventional APD with constant volumes and dwell times. These benefits of aAPD have subsequently been confirmed in a randomized crossover trial in adults published in 2011 [6]. In this study, patients received 45 days of aAPD consisting of 2 small, 45-minute dwells of 1500 mL followed by 3 large, 150-minute dwells of 3000 mL, or conventional APD comprising 6 cycles of 2000 mL over 90 minutes each, in randomized order. Despite identical duration of overnight APD and total dialysate volume, aAPD significantly improved ultrafiltration volume and efficiency, increased urea, creatinine, and phosphate removal by about 10%, and almost doubled sodium removal per mL of ultrafiltration achieved compared to conventional APD, additionally resulting in improved blood pressure control. Similar promising results regarding ultrafiltration, Kt/V_urea_, creatinine clearance, sodium extraction and glucose absorption were subsequently observed in 2 small aAPD trials in Spain and France [7, 8].

While the aAPD scheme investigated by Fischbach et al. [6] included dwells with the same volumes and durations for all study participants, the authors also indicated that an individualized adaption of time and volume for short and long dwells according to clinical evaluation may result in an even greater benefit. A personalized aAPD concept was published by Galli et al. in 2011 [9]. By using programmed prescriptions determined by computer simulation, treatments were adjusted to the individual patients’ peritoneal transport status while taking into account their residual diuresis and need for ultrafiltration. Individualization of the APD treatment improved weekly peritoneal Kt/V and creatinine clearance without affecting renal clearance. The mean total Kt/V exceeded the target values set out in the U.S. National Kidney Foundation Kidney Disease Outcomes Quality Initiative (NKF-KDOQI) clinical practice guidelines for peritoneal dialysis adequacy [10, 11]. Importantly, both individualized therapeutic approaches improved dialysis efficacy while requiring either minimal hypertonic PD solutions (>1.5% dextrose) or none at all, without substantial increases in APD duration. Higher infusion and tidal volumes may partly explain the improvements with the individualized prescriptions of Galli et al. [9] whereas total dialysate volume remained unchanged in the aAPD trials of Fischbach et al. [5, 6].

We report on the results of the Peritoneal Dialysis—Improved Dialysis Efficiency with Adapted APD (PD-I.D.E.A.) study carried out in a multinational, prospective and non-interventional (observational) design that was performed to assess the volume status of a large cohort of aAPD patients and to observe its development during one year using bioimpedance spectroscopy (BIS). Secondary objectives were to follow up additional patient outcomes such as residual renal function (RRF), dialysis prescription over time, change of therapy modality, achievement of adequate solute removal, and therapy tolerability.

## Methods

### Study design and schedule

PD-I.D.E.A. was designed as a non-interventional, prospective, international multicenter study. Study procedures started with a baseline examination during which the patients’ eligibility for participation was determined and informed consent was obtained. Basic patient, medical history, and treatment data were taken from the patient files. Follow-up data were documented during site visits performed about every 3 months according to local practice, for a total period of about 1 year, or until a patient was withdrawn from the study due to transfer to HD, kidney transplantation or any other reason.

### Participants

Patients with chronic kidney disease (CKD), ≥18 years of age, with an indication for kidney replacement therapy were eligible for participation if they were already on aAPD treatment or about to be treated, using a sleep•safe or sleep•safe harmony PD cycler (Fresenius Medical Care, Bad Homburg, Germany). Moreover, regular routine monitoring of the volume status by means of the Body Composition Monitor (BCM; Fresenius Medical Care, Bad Homburg, Germany), a validated medical device applying bioimpedance analysis [12] was required. To avoid selection bias, participating sites were instructed to include consecutive patients who met the selection criteria.

### Treatments

In this non-interventional study, all treatment decisions were at the discretion of the attending physician who prescribed the aAPD regimen in accordance with their best clinical judgment. For nightly aAPD as well as for daytime dwells, the number of exchanges as well as the solution, the glucose concentration, the inflow volume, and the dwell time prescribed and actually administered were recorded. There were no restrictions for concomitant treatment.

### Ethical conduct

The study was performed in accordance with the Declaration of Helsinki, the ISO 14155:2011 Good Clinical Practice guideline, as well as with the respective European and national laws and regulations. Where required, the approval of the observational plan by an independent ethics committee was obtained. All participants provided written informed consent.

The study was registered prospectively at ClinicalTrials.gov under identifier NCT02470598.

### Outcomes

Outcomes assessed during all visits included absolute and relative fluid overload, body composition parameters, dialysis outcomes (renal, peritoneal, and total creatinine clearance and Kt/V, 24-h urine output, mean daily ultrafiltration, total 24-h output, sodium excretion), peritoneal membrane transport status (if available), vital signs, and laboratory measures in blood, dialysate, and urine. Safety was assessed based on incident reports.

Data were documented using a secured, internet-based electronic data capture system. Volume status and body composition were determined non-invasively using the BCM device.

### Statistical analysis

All outcomes were analyzed descriptively. For serial measurements, mixed models for repeated measures (MMRM) were applied for analyzing time courses of observed data as well as intraindividual differences to baseline (using the baseline value as a covariate). For these outcomes, all reported mean values and their 95% confidence intervals (CIs) are marginal means and associated CIs from the MMRM analyses. Due to the nature of CKD as a chronic, progressive disorder, a certain proportion of patients were expected to withdraw from the study before the scheduled end of the follow-up period. To assess the impact of early withdrawals on the results of the longitudinal analyses, pattern mixture models [13] were fitted as sensitivity analyses.

For longitudinal outcomes, subgroup analyses were performed to compare aAPD prevalent vs. incident patients, subsets defined by different aAPD treatment patterns, by total dwell time, by PD solution, by glucose concentration, and by country.

Participants were classified as PD or aAPD incident when they had been on this treatment for up to 7 days at baseline, and as PD or aAPD prevalent otherwise. aAPD dwells were categorized as ‘small’ for inflow volumes <70% of the maximum prescribed volume, as ‘large’ for volumes ≥70% of the maximum, and as ‘constant’ when all dwells of an aAPD prescription were ≥70% of the maximum. Likewise, dwells were classified as ‘short’ for dwell times <70% of the maximum prescribed time, as ‘long’ for dwell times ≥70% of the maximum, and as ‘constant’ when all dwells of a prescription were ≥70% of the maximum. For fluid overload, patients were considered underhydrated or overhydrated if their values were below the 10^th^ or above the 90^th^ percentile of a presumed healthy reference population, i.e. for absolute volumes < -1.1 L or > +1.1 L or for a relative overload < -7% or > +7% relative to extracellular water [14, 15].

The analyses were based on all patients with baseline data and any post-baseline data. For longitudinal analyses, patients with missing baseline data for the outcome of interest were excluded. All reported p-values are intended for purely descriptive interpretation.

## Results

### Participant accountability and baseline characteristics

Between April 2015 and June 2018, a total of 180 patients were recruited in 48 centers. In accordance with the clinical observational plan, 20 patients were excluded from the analysis due to either no valid baseline BCM measurement (n = 4), no completed baseline visit (n = 12), BCM measurement performed more than 30 days prior to or after enrolment (n = 2), or loss to follow-up immediately after baseline (i.e., without any post-baseline data). Thus, 160 patients from 39 centers in Spain (n = 53), Malaysia (n = 50), the Czech Republic (n = 35), Sweden (n = 14), Denmark (n = 4), the Netherlands (n = 3), and Finland (n = 1) could be analyzed. Each site contributed between 1 and 19 patients to the analysis population. The number of patients per center is shown in Table 1.

**Table 1:** Number of patients per center (analysis population).

Centers with …	No. Centers	No. Patients
1–5 patients	30 (76.9%)	75 (46.9%)
6–10 patients	7 (17.9%)	54 (33.8%)
>10 patients	2 (5.1%)	31 (19.4%)
Total	39 (100.0%)	160 (100.0%)

**Fig 1:**
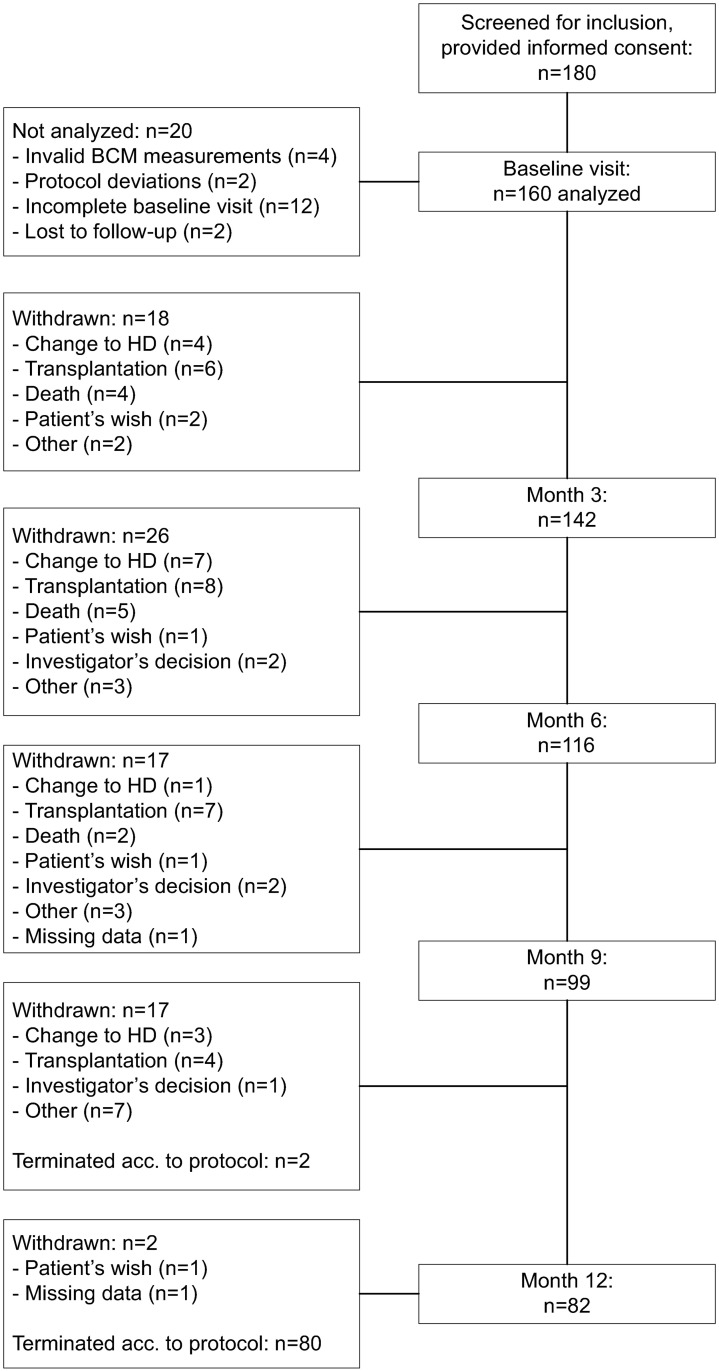
Disposition of patients.

A total of 82 patients (51.3% of 160) completed the study as scheduled after about 12 months (Fig 1). Known reasons for withdrawal were transplantation (15.6%), change to HD (9.4%), any-cause death (6.9%), for which cardiac failure was the predominant cause, patient’s or investigator’s wish (6.3%). Two patients (1.3%) were lost to follow-up, and for 15 patients (9.4%) the reason for withdrawal was specified as ‘Other’ without indicating any details. Identified reasons for transfer to HD were recurrent infection (6/15 patients), ultrafiltration failure (2 patients), inadequacy, and patient’s wish (1 patient each). Baseline data are summarized in Table 2.

**Table 2:** Baseline characteristics (mean ± SD or number and %; analysis population).

	Valid n	Result
**Age (years)**	160	57.7 ± 14.1
**Sex: female**	160	67 (41.9%)
**Body weight (kg)**	149	72.9 ± 18.1
**Body height (cm)**	160	166 ± 10
**Body mass index (kg/m^2^)**	149	26.4 ± 5.3
**Body surface area (m^2^)**	149	1.82 ± 0.26
**Vital signs**		
• Systolic blood pressure (mm Hg)	160	139.2 ± 21.4
• Diastolic blood pressure (mm Hg)	160	79.7± 11.6
• Heart rate (beats/minute)	153	75.7 ± 12.7
**Underlying renal disease**	160	
• Diabetes mellitus (type 1 and 2)		44 (27.5%)
• Hypertensive / large vessel disease		32 (20.0%)
• Glomerulonephritis		28 (17.5%)
• Cystic / hereditary / congenital diseases		14 (8.8%)
• Other or unknown		40 (25.0%)
**Comorbidities**	160	
• Diabetes (type 1 and 2)		96 (60.0%)
• Hypertension		152 (95.0%)
**History of peritonitis**	160	28 (17.5%)
**Age-adjusted Charlson comorbidity index**	160	4.6 ± 1.8
**Medication (%)**	160	
• Antihypertensives		128 (80.0%)
• Diuretics		114 (71.3%)
• Phosphate binders		115 (71.9%)
**Volume status and body composition**		
• Fluid overload, absolute (L)	149	1.8 ± 2.9
• Overhydrated patients acc. to absolute fluid overload	149	80 (53.7%)
• Fluid overload, relative (%)	140	9.9% ± 14.0%
• Overhydrated patients acc. to relative fluid overload	140	79 (56.4%)
• Lean tissue mass (kg)	146	35.6 ± 13.5
• Adipose tissue mass (kg)	145	30.2 ± 16.0
**Residual renal function and dialysis efficiency**		
• 24h urine output (mL)	146	1042 ± 771
• Weekly renal creatinine clearance (L)	108	50.6 ± 48.5
• Weekly peritoneal creatinine clearance (L)	99	30.9 ± 13.2
• Weekly peritoneal Kt/V	96	1.4 ± 0.5
• Weekly renal Kt/V	97	0.7 ± 0.6
• Mean daily ultrafiltration (mL)	150	645 ± 619

Time on PD ranged between 0 and 9.8 years, with a median vintage of 1 year. Sixty-five patients (40.6%) were aAPD incident, with an aAPD vintage of 7 days or less, while the remaining 59.4% were aAPD prevalent. Mean (± SD) baseline intra-peritoneal pressure (IPP) was 16.1 ± 5.4 cm H2O, and 32/106 patients with valid data (30.2%) had a baseline IPP >18 cm H_2_O. Out of 110 patients with a valid peritoneal transport status test at baseline, 47 (42.7%) were in the low or low average range and 63 (57.3%) were in the high average or high range.

### aAPD prescription and treatment

**Fig 2:**
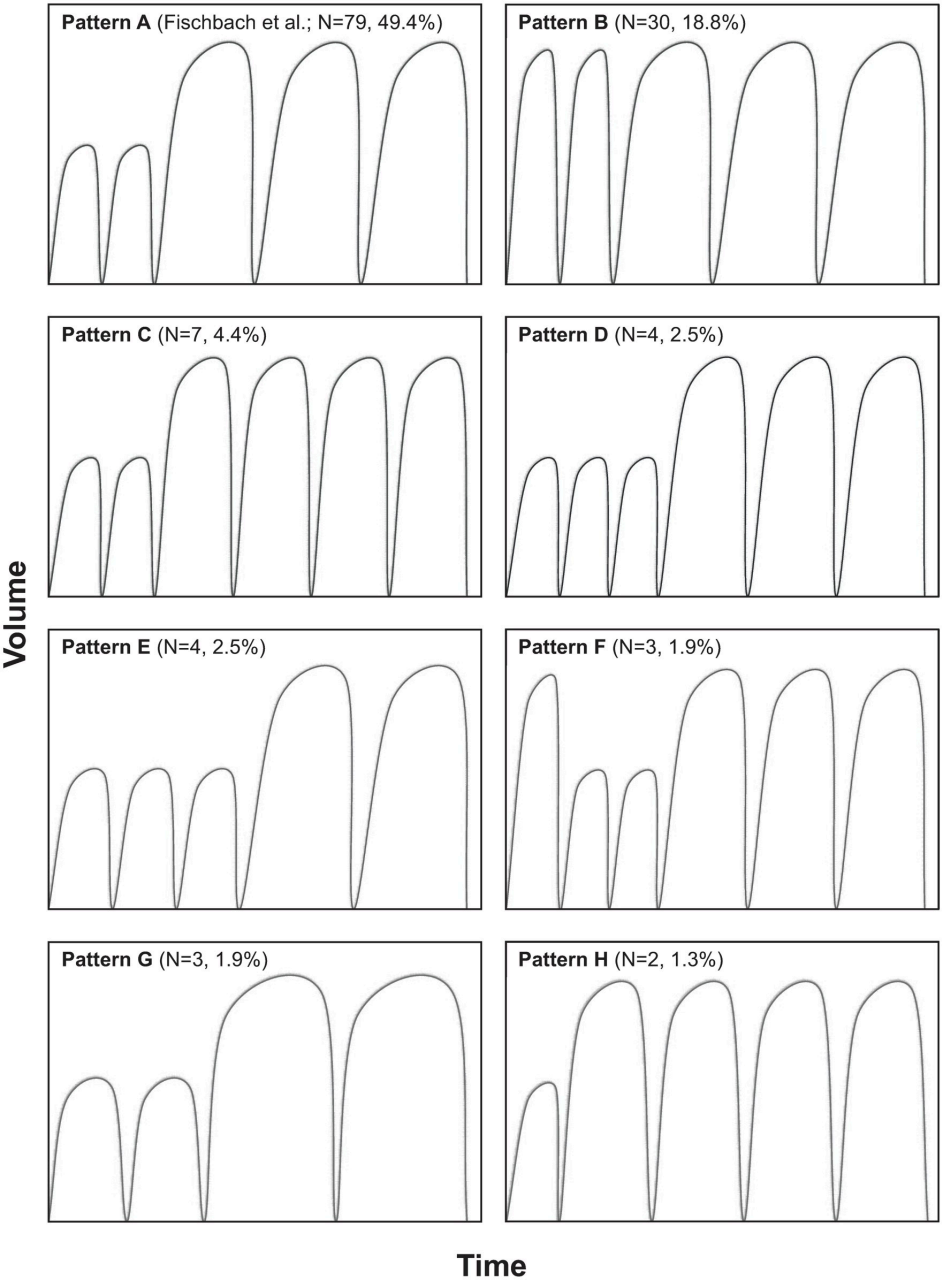
aAPD patterns prescribed in more than 1 patient at baseline (note that the panels are intended to visualize the sequences of ‘short’ and ‘long’ and of ‘small’ and ‘large’ dwells rather than specific dwell times and volumes).

A total of 33 different APD treatment patterns were prescribed, out of which 6 either included only constant volumes and dwell times, or were used in patients excluded from further analysis as shown in Fig 1. In the patients analyzed, 27 different aAPD patterns were thus observed. Fig 2 shows all patterns that were observed in more patients than 1 at baseline. Pattern A, 2 small, short dwells followed by 3 large, long dwells, corresponds to the pattern recommended by Fischbach and colleagues (6) and was used by 79 patients (49.4%), followed by patterns B (30 patients, 18.8%) and C (7 patients, 4.4%). All other patterns were used in less than 5% of the participants, and 19 of the 27 patterns were prescribed to only a single patient. The number of prescribed dwells ranged between 4 and 9 per night, with about 80% of the patients receiving 5 dwells, and 16.3% receiving more than 5 dwells.

Mean baseline dwell times ranged between 40 and 123 min. (mean ± SD: 77 ± 16 min.), with averages of 39 ± 11 min. for short dwells and of 103 ± 26 min. for long dwells. Total aAPD treatment time ranged between 166 and 740 min. (396 ± 83 min.), with 87 patients (54.4%) having total aAPD dwell times of <7 hours at baseline and throughout the entire follow-up. Individual aAPD total inflow volumes at baseline ranged between 7000 and 13750 mL (9908 ± 1561 mL) absolute, or between 593 and 1,739 mL/m^2^ (1060 ± 156 mL/m^2^) body surface area. About 60% used a non-hypertonic PD solution (1.5% dextrose). At baseline, 39 patients (24.5%) had a wet day in addition to aAPD, with similar proportions of patients with daytime exchanges throughout the follow-up.

During the 1-year follow-up period, 19 patients (11.9.%) had a change in the prescribed aAPD pattern, 26 (16.3%) changed from a total aAPD dwell time of <7 hours to ≥7 hours or vice versa, 23 (14.4%) changed from a dry day to a wet day or vice versa while the total proportion of patients with daytime exchanges remained stable at about 25% during the observation period. Major discrepancies between prescription and actual treatment were observed in less than 13% of the patients per visit. The most frequent discrepancies were differences in the number of exchanges by >10% of the prescription and the use of a non-prescribed solution.

### Volume status, arterial blood pressure, and body composition

**Fig 3:**
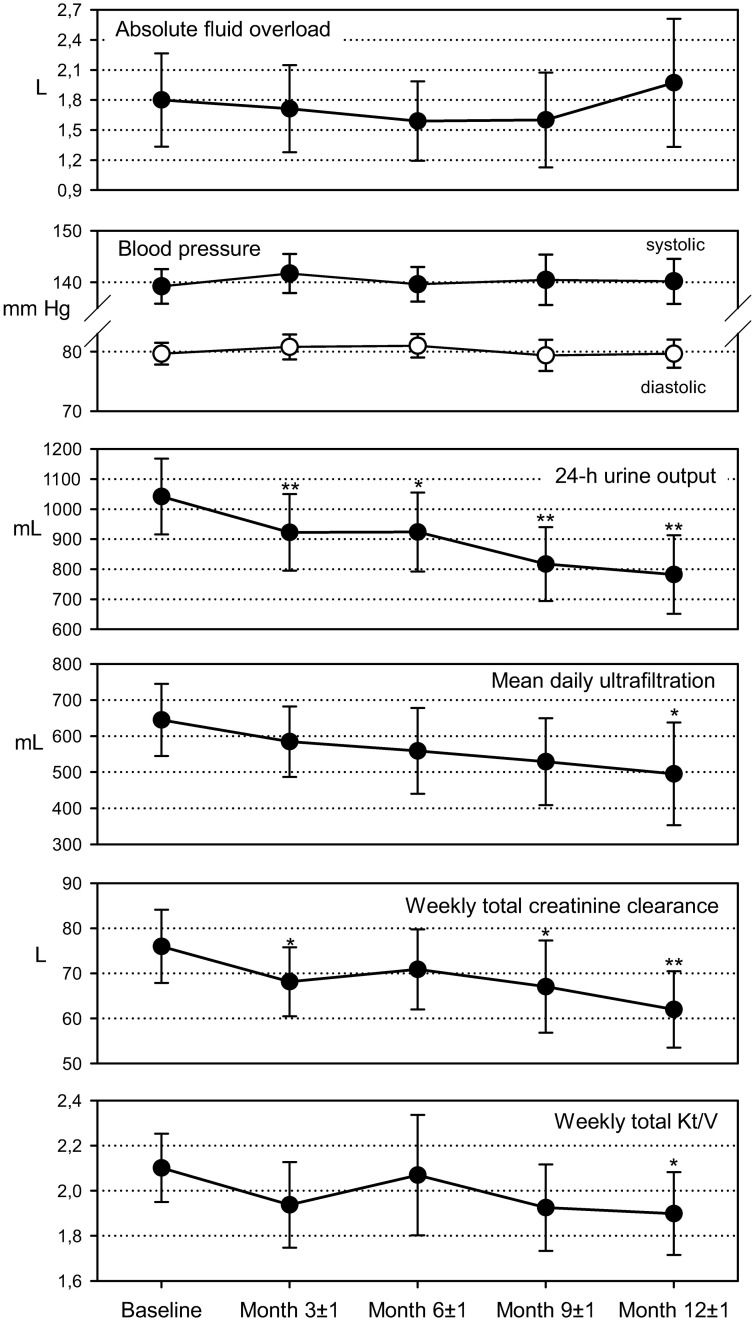
Time courses of hydration, residual renal function, and toxins removal parameters (marginal means and 95% confidence intervals from mixed models for repeated measures, number of patients with valid data). Differences to baseline: *—p<0.05; **—p<0.01.

The time course of absolute fluid overload showed no uniform trend towards increasing or decreasing overhydration, with marginal means ranging between 1.59 (95% CI: 1.19; 1.99; month 6) and 1.97 (1.33; 2.61; month 12) L (Fig 3). Mean intra-individual differences to baseline ranged from a 0.32 (0.01; 0.62) L decrease at month 6 to an 0.05 (-0.55; 0.65) L increase at month 12. For relative fluid overload, the proportion of euvolemic patients changed only marginally, with 36.4% at baseline to 36.2% after one year, and there were also only marginal changes in the the proportion of overhydrated patients, with 56.4% and 56.5%, respectively. Fig 3 also show only very limited variation of arterial blood pressure over time, with marginal mean in a range between 139.2 (135.9; 142.6; baseline) and 141.7 (136.3; 145.5; month 3) mm Hg for systolic pressure and between 79.4 (76.8; 82.0; month 9) and 81.0 (79.0; 83.0; month 6) mm Hg for diastolic pressure.

Body composition measurements such as body mass index, lean tissue index, and fat tissue index changed only marginally throughout the follow-up period, with changes from baseline never reaching statistical significance.

### Residual renal function and uremic toxins removal

We observed a gradual deterioration of RRF, ultrafiltration and uremic toxins removal throughout the observation period, with descriptively significant decreases versus baseline for the outcomes shown in Fig 3. Mean value decreases at 1 year were 259.4 (148.3; 370.5) mL for 24-hour urine output, 149.5 (6.8; 292.1) mL for mean daily ultrafiltration, 14.0 (5.7; 22.3) L for weekly total creatinine output, and 0.20 (0.02; 0.39) for weekly total Kt/V. Deterioration of total creatinine clearance and Kt/V were mainly driven by decreases in renal clearance and Kt/V, respectively, and only to a lesser degree by changes in peritoneal clearance and Kt/V. Sodium removal decreased gradually, from 1.074 (1.066; 1.094) mmol/day at baseline to 1.047 (1.019; 1.076) mmol/day after 1 year (p = 0.077).

Patients with shorter total aAPD dwell times (<7 hours) tended to exhibit a more favorable RRF than those with longer dwell times. Otherwise, subgroup analyses performed for selected hydration, kidney function, and dialysis efficiency parameters revealed partly large, statistically important baseline differences between the pre-defined subsets so that conclusive comparisons between the subset time courses of longitudinal outcomes could not be obtained.

### Differences between countries

A subgroup analysis by country was performed. The analysis compared patients from the Czech Republic, Malaysia, Spain, and the pooled data from all other countries with regard to parameters of volume status, residual renal function, and dialysis efficiency. The main results are shown in Table 3. Change between baseline and month 6 was chosen for presentation because data for the second quarterly follow-up period were still available for about ^2^/_3_ of the included patients.

**Table 3:** Subgroup comparison by country for parameters of volume status, residual renal function, and dialysis efficiency—Baseline value and intra-individual change between baseline and the end of follow-up month 6 (mean ± SD, and number of patients with valid data in the analysis population).

Outcome		Czech Republic	Malaysia	Spain	All other countries
Fluid overload (L)	Baseline	1.63 ± 1.89 n = 34	2.92 ± 3.99 n = 45	0.82 ± 2.03 n = 50	2.02 ± 2.31 n = 20
	Change	-0.12 ± 2.06 n = 19	-0.12 ± 2.16 n = 34	+0.08 ± 1.49 n = 22	-0.02 ± 1.59 n = 10
24-hour urine output (L)	Baseline	1.37 ± 0.78 n = 34	0.62 ± 0.47 n = 43	1.13 ± 0.77 n = 51	1.17 ± 0.95 n = 18
	Change	-0.44 ± 0.54 n = 16	-0.10 ± 0.43 n = 19	-0.27 ± 0.63 n = 25	-0.46 ± 0.67 n = 7
Mean daily ultrafiltration (mL)	Baseline	828 ± 478 n = 35	405 ± 638 n = 41	723 ± 474 n = 52	615 ± 919 n = 22
	Change	-42 ± 229 n = 18	-62 ± 895 n = 33	-68 ± 869 n = 25	+210 ± 532 n = 12
Total output (L)	Baseline	2.16 ± 7.45 n = 35	0.89 ± 0.81 n = 49	1.79 ± 0.85 n = 53	1.57 ± 1.04 n = 22
	Change	-0.42 ± 0.59 n = 18	-0.40 ± 1.10 n = 37	-0.45 ± 0.93 n = 27	-0.30 ± 0.76 n = 12
Weekly total creatinine clearance (L)	Baseline	80.3 ± 34.9 n = 18	58.5 ± 26.1 n = 33	86.0 ± 46.1 n = 33	107.1 ± 19.9 n = 6
	Change	+2.6 ± 35.3 n = 10	-8.1 ± 24.1 n = 18	-10.2 ± 38.9 n = 16	-19.8 ± 35.5 n = 3
Weekly total Kt/V	Baseline	2.10 ± 0.71 n = 18	1.96 ± 0.73 n = 33	2.13 ± 0.64 n = 27	2.59 ± 0.53 n = 5
	Change	+0.04 ± 0.87 n = 9	-0.19 ± 0.04 n = 17	+0.30 ± 1.34 n = 13	+0.04 ± 0.26 n = 2
Sodium removal (mmol/day)	Baseline	1.08 ± 0.02 n = 11	0.97 ± 0.08 n = 7	1.10 ± 0.06 n = 29	1.04 ± 0.06 n = 6
	Change	+0.01 ± 0.07 n = 7	+0.07 ± 0.06 n = 6	-0.02 ± 0.09 n = 15	0.00 ± 0.08 n = 2

For hydration and residual renal function parameters, Table 3 shows noteworthy baseline differences, mainly between patients from Malaysia on one hand and patients from other countries on the other (on average, patients from Malaysia had more severe volume overload and less residual renal function). Change from baseline, on the other hand, was mainly comparable for all countries. For some of the parameters the interpretation of the results is complicated by the low number of patients with valid data.

### Sensitivity analyses

The impact of missing data on the results of longitudinal outcomes was analyzed by fitting pattern mixture models for absolute fluid overload, mean daily ultrafiltration, and weekly creatinine clearance. Patterns were defined by the individually last visit attended by a patient.

**Fig 4:**
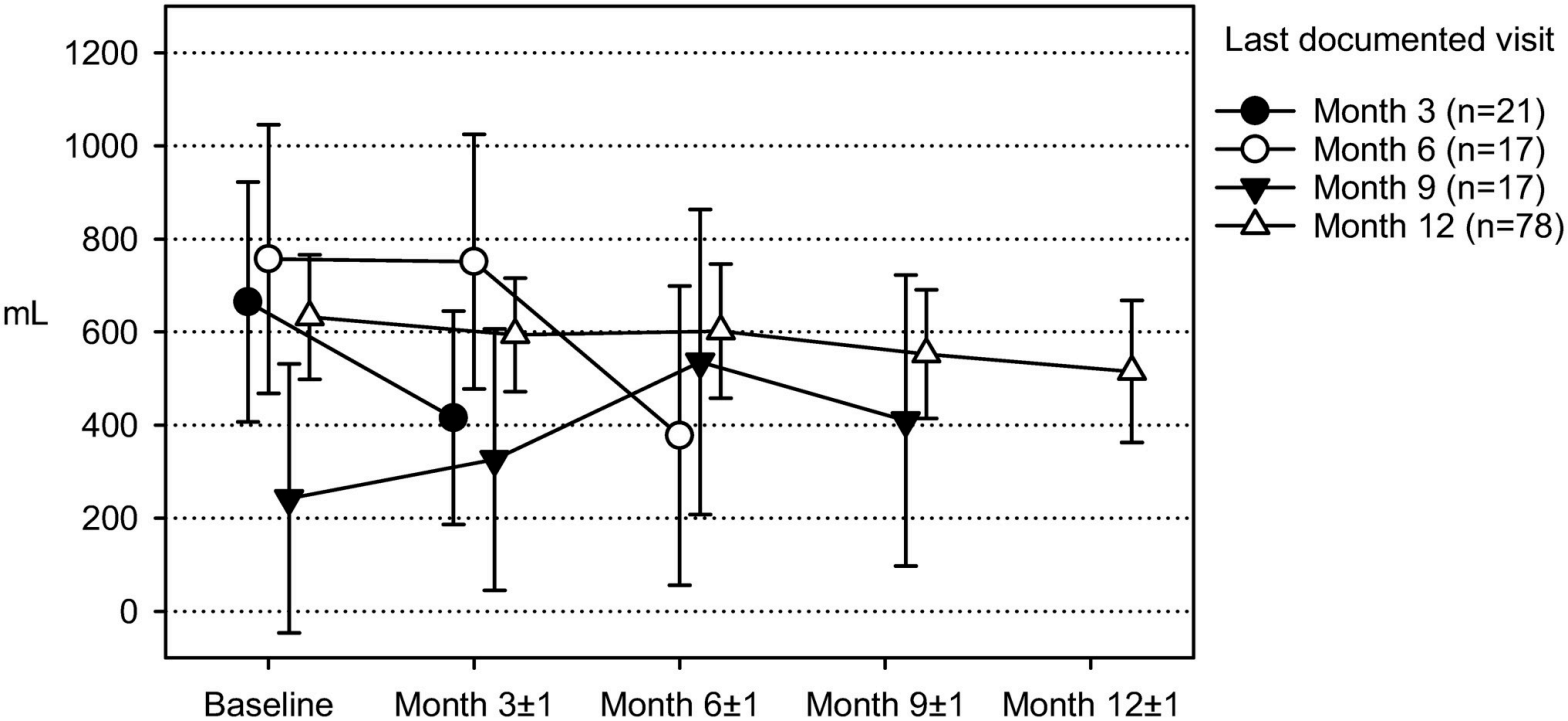
Mean daily ultrafiltration—Time course of subsets of patients terminating their study participation at different visits (marginal means and 95% confidence intervals from mixed model for repeated measures).

As an example, Fig 4 shows the results for mean daily ultrafiltration. The trajectories in the figure represent subsets of patients who dropped out at months 3, 6, or 9, or who completed the study as scheduled after 12 months. The trajectories of all dropout subsets show a notable decrease in ultrafiltration during the period before the last documented visit. Study completers, on the other hand, also showed a gradual but less pronounced decline in ultrafiltration but no accelerated deterioration. Linear mixed model analyses performed for each withdrawal pattern showed significant pattern by visit interactions (p<0.05) for patients whose last visits were documented at months 6 or 9.

### Safety assessments

Across the 1-year follow-up, 35/142 (24.6%) of the patients who attended at least one post-baseline visit reported peritonitis at least once.

A total of 6 events potentially device related incidents were reported for 5/160 patients (3.1% of 160). Technical issues were failure to store the data of a treatment due to battery outage, device leakage, and air in the tubing system, probably caused by an operating error, that set off an alarm. Moreover, 3 episodes of peritonitis requiring or prolonging hospitalization were described as potentially device-related. No incident was fatal.

## Discussion

About 2 ½ decades ago, aAPD was introduced with the aim of coping with the major challenges of ‘traditional’ PD, to provide adequate volume control while optimizing sodium and uremic toxins removal [16]. This non-interventional research assessed the application of aAPD in clinical routine care, including current prescription practices and dialysis effectiveness.

For aAPD prescriptions, participating nephrologists adhered to the aAPD pattern proposed by Fischbach and colleagues (6, 16; 2 short, small dwells followed by 3 long, large dwells) short, small dwells followed by 3 long, large dwells) for nearly half of the documented cases, and a very similar pattern in which the initial 2 short, small dwells were replaced by 2 short, large dwells were used in another 19% of patients. The results nevertheless indicate a great diversity of aAPD prescriptions in clinical practice, with a total of 27 different patterns, different fill volumes and dwell times, and with varying PD solutions and glucose concentrations. During the 1-year follow-up, we also observed modifications in aAPD pattern, total treatment time or fill volume, PD solution and/or the addition or discontinuation of daytime exchanges in an appreciable proportion of patients, indicating that nephrologists were attempting to further optimize their individualized prescriptions, notably in patients who were aAPD incident at baseline.

Given the large number of different aAPD patterns documented in this study, it is difficult to draw conclusions on specific patterns. Indeed, in subgroup analyses by aAPD pattern performed on measures of volume status and RRF, no systematic differences could be observed. It should, however, be considered that the underlying principle of aAPD is individualized optimization, and if the treatment regimens of the study participants are assumed to have been individually optimized, then no systematic differences between aAPD patterns are to be expected.

Even though aAPD has meanwhile been used for about 25 years, results from systematic research regarding the appropriate total treatment time are still sparse. Fischbach and colleagues used a total treatment time of 540 min. [6, 17] which is consistent with a recently published guidance document for APD in volume overloaded patients that recommends a 9-hour total treatment period with a maximum of 5 overnight cycles in high (fast) and high-average and a maximum of 4 cycles in low and low-average transporters [18]. Among the participants of this study, the average prescribed treatment times were considerably shorter, with more than half of the patients treated for 7 hours or less. These shorter treatment times mainly reflect the degree of RRF and a lower need for peritoneal clearance in incident patients when entering the study, when longer dwells might have contributed to a more rapid progression of RRF decline. In this context, it is, however, also worth mentioning that for about ^1^/_3_ of the study participants no peritoneal transport status test results were available at baseline for verifying whether the prescribed dwell times were in line with the recommendations. Data from the literature suggest that many nephrological centers tend to perform this test only annually and sometimes even at longer intervals [2, 4]. It may, however, be advisable to test the patients’ peritoneal transport status more frequently in order to be able to individually adjust the parameters of aAPD accordingly.

Due to the time required for filling and draining, too rapid exchanges limit active treatment time and thus bear the risk of overhydration and sodium sieving [18]. Whereas in the early phase of the dwell mainly free water is removed via aquaporin-1 channels, removal of sodium-coupled water occurs only later during the dwell via the small pores of the peritoneum, a process which is essential for adequate sodium and water balance and for blood pressure control [19, 20]. Adequate duration of the long dwells is thus equally important to contribute to total ultrafiltration by sodium-coupled water transport and to remove uremic toxins such as phosphate in sufficient amounts [16]. Considering the comparatively short total dwell times in this study and the fact that 16% of the patients received more than 5 dwells per aAPD treatment, it is worth investigating whether dwell time per exchange could have been sufficient to achieve adequate ultrafiltration and solute clearance.

Optimization of solute clearance requires large dwells which promote uremic toxin removal both due to a greater diffusion volume and recruitment of a larger peritoneal surface area [16, 21–23]. Based on their observation that intolerance of intraperitoneal volume was associated with an increase of IPP above 20 cmH_2_O in adults, Durand et al. suggested an IPP threshold of 18 cm H_2_O in the supine position to determine the maximum tolerable volume [24, 25]. In the study sample, IPP measurements were only available for approximately ^2^/_3_ of the patients out of which close to ^1^/_3_ had an IPP >18 cm H_2_O at baseline, indicating that intraperitoneal volume in these patients may have been higher than recommended.

The observation that only about ¼ of the analyzed patients received daytime exchanges is quite unusual. In this non-interventional study, any treatment decisions were strictly at the discretion of the attending physicians, and unfortunately no explanation could be derived from the documented data as to why daytime exchanges were not more widely prescribed.

In the majority of patients, parameters of volume status and body composition were stable across the 1-year follow-up. Otherwise, the results point to a gradual deterioration of renal functioning and uremic toxins removal, with decreasing urine output, ultrafiltration, and total output as well as with decreasing renal and total creatinine clearance and Kt/V. These observations are consistent with the fact that almost half of the study participants did not complete the 12-month observation period as scheduled while the 3 most frequently reported reasons for premature withdrawal were renal transplantation, change to HD, and death, together accounting for more than 70% of drop-outs, all of which are more or less indicative of a progression of CKD. The results are thus likely to reflect the natural course of the underlying renal disease. The main reasons for withdrawal from our study are consistent with those reported elsewhere for continuous ambulatory peritoneal dialysis (CAPD) or APD. For example, in a 3-year observational study with incident CAPD and APD patients, the main dropout reasons were transfer to HD and transplantation, which occurred in 23% and in 22% of the patients, respectively, and accounted for about ^2^/_3_ of all dropouts [2]. Moreover, in both studies, the main reason for transfer to HD was infection.

The interpretation of the serial measurements of volume status, total fluid output, and body composition, and uremic toxins removal data is complicated by the gradual withdrawal of patients from the study, mainly for reasons that were, as already indicated, likely associated with a deterioration of CKD. This interpretation is supported by the results of our sensitivity analyses which indicate that missing data due to patient withdrawal were informative rather than at random: as illustrated in Fig 4, patients who did not complete the 12-month observation period tended to suffer from a notable deterioration of their RRF during the last 3 months before dropping out, and consequently the main dropout reasons were renal transplantation, change to HD, and death as already mentioned. These sensitivity analyses also indicate, however, that patients who completed the study as scheduled were mainly in a stable condition throughout the 1-year follow-up so that no clinically meaningful deterioration occurred under the prescribed aAPD regimen.

For many of the outcomes, prospectively defined subgroup analyses revealed more or less substantial baseline differences between subsets. Since the majority of patients were aAPD prevalent (or at least PD prevalent) at study inclusion, it cannot be determined whether the status of a patient captured at baseline led the participating nephrologists to choose the features of the individualized aAPD treatment regimen that were at the same time the criteria which defined the subgroups (e. g., aAPD pattern, dwell time, glucose concentration), or whether the baseline differences were rather the result of the aAPD treatment already received before baseline. In a non-interventional design that includes both incident and prevalent patients, such limitations can hardly be avoided.

Another limitation of the study is the fact that the reasons for choosing a specific aAPD regimen for a particular patient were not fully solicited. Comprehensive information about how, and based on which observations, the parameters of aAPD prescriptions are determined in clinical practice would, however, increase the understanding of aAPD in routine care.

While the study was performed mainly to collect information on current practices in aAPD during every-day clinical routine, the uncontrolled, observational design of the project does not permit causal inference.

In conclusion, the results indicate that the therapeutic options available in aAPD using a Fresenius sleep•safe or sleep•safe harmony PD cycler are actually being used by practitioners to individualize their patients’ dialysis prescriptions. While the non-interventional design of the study necessarily imposes limitations on the assessment of treatment efficacy, the time courses of the renal function and dialysis efficiency measures investigated were consistent with what one may expect in a population of patients suffering from chronic, progressing renal disease. Moreover, no evidence was observed that the individualization of the aAPD options performed by the participating nephrologists may have caused any adverse effects.
